# Quadriceps force and medio-laterally directed joint force during knee flexion in a personalized patellofemoral joint model

**DOI:** 10.1186/s12891-025-09397-y

**Published:** 2025-12-12

**Authors:** Annabelle Weigert, Leandra Bauer, Hanna Jacobi, Matthias Woiczinski, Antje Dinauer, Boris M. Holzapfel, Peter E. Müller, Thomas R. Niethammer

**Affiliations:** 1 Department of Orthopedics and Trauma Surgery, Musculoskeletal University Center Munich (MUM), University Hospital, LMU Munich, Munich, Germany; 2 Experimental Orthopedics, Campus Eisenberg, Waldkliniken Eisenberg, University Hospital Jena, Friedrich-Schiller-University Jena, Jena, Germany

**Keywords:** Patellofemoral instability, Musculoskeletal modeling, MRI-based simulation, Trochlear dysplasia, TT–TG distance, Squat analysis, AnyBody modeling system, Patient-specific biomechanics

## Abstract

**Background:**

Patellofemoral instability (PFI) is a multifactorial condition influenced by complex interactions between anatomical structures and dynamic stabilizers. Accurate assessment of mediolateral patellofemoral joint loading remains challenging, particularly during physiologically relevant joint angles simulated under controlled kinematic conditions. Anatomy-based individualized musculoskeletal models provide an in-silico approach to investigate these biomechanical parameters, such as the loading patterns. The purpose of this study was to develop individualized musculoskeletal knee models based on MRI-derived anatomical data from cadaveric specimens and to quantify the mediolateral component of the patellofemoral joint reaction force during a standardized, computer-driven squat simulation. Quadriceps muscle forces were estimated to assess the demand on dynamic stabilizers.

**Method and material:**

: MRI scans from four cadaveric lower limbs were segmented to reconstruct the distal femur, proximal tibia, and patella. The resulting STL bone models of the femur, tibia, and patella were aligned to TLEM2 templates using anatomical landmarks in CATIA V5 and integrated into the AnyBody Modeling System via custom AnyScript routines. A predefined, computer generated squat motion consisting of asymmetric flexion–extension cycle between 0° and 90° at a constant, scripted angular velocity of 60°/s was applied using a kinematic driver. The original bodyweight-level loading conditions from the AnyBody squat model were retained; no external weights or EMG data were included. Patellofemoral joint reaction forces and quadriceps muscle forces were estimated using inverse dynamics calculations.

**Results:**

The mediolateral patellofemoral joint reaction force increased with knee flexion in all models, reaching specimen-specific peaks ranging from 7 N/kg BW to 67 N/kg BW (maximum of 24 ± 25 N/kg BW). The model exhibiting a pronounced supratrochlear bony prominence (Dejour type B trochlear dysplasia) showed the highest lateral loading and quadriceps force. Muscle forces are reported as magnitudes.

**Conclusion:**

This study demonstrates that MRI-derived, individualized knee geometries can be integrated into a musculoskeletal simulation framework. This allows the investigation of how anatomical variation affects patellofemoral joint loading during a controlled, computer-generated squat motion. By integrating individualized knee anatomy into a validated simulation framework, our approach enables the in-silico analysis of anatomical risk factors—such as trochlear dysplasia and increased TT–TG distance—that are clinically relevant for patellofemoral instability. This method may support future biomechanical investigations and preoperative planning by providing reproducible, anatomy-driven insights into joint mechanics.

## Introduction

Patellofemoral instability (PFI) is a multifactorial clinical condition characterized by recurrent episodes of patellar subluxation or dislocation, often associated with anterior knee pain and functional limitations. Its etiology involves a combination of osseous and soft-tissue abnormalities, including trochlear dysplasia, patella alta, increased tibial tubercle–trochlear groove (TT–TG) distance, valgus alignment, torsional deformities, and generalized ligamentous laxity [1–4]. These anatomical variations can disrupt normal patellar tracking and contribute to instability by altering load distribution across the patellofemoral joint.

Patellar tracking is governed by a complex interaction between passive (bony and ligamentous) and active (muscular) stabilizers. During early knee flexion, stability relies primarily on soft-tissue constraints such as the medial patellofemoral ligament, whereas at greater flexion angles, bony engagement with the trochlear groove becomes increasingly important [5–7]. Additionally, an increased Q-angle or lateralized TT–TG distance can induce excessive lateral forces on the patella, promoting maltracking and joint degeneration [8–10].

Although clinical and radiological assessment can identify some of these risk factors, it remains difficult to predict how specific anatomical features influence patellofemoral joint loading during dynamic movement. A deeper biomechanical understanding is essential for developing patient-specific treatment strategies.

The present study addresses this challenge by incorporating MRI-derived knee geometry into individualized musculoskeletal model using the AnyBody Modeling System. A previously validated squat simulation model was adapted to evaluate how anatomical variation affects patellofemoral joint reaction forces. The primary objective was to quantify the mediolateral force component of the patellofemoral joint reaction force during a standardized, computer-generated squat motion. This analysis aims to characterize loading patterns that may contribute to lateralization tendencies of the patella and patellofemoral instability.

## Materials and methods

In this context, the present study employed the AnyBody Modeling System (AnyBody Technology A/S, Aalborg, Denmark). This software enables the creation and analysis of human body models, incorporating bones, muscles, tendons, joints, and ligaments. To this end, AnyBody provides a library of various musculoskeletal body models along with corresponding application examples [11].

### MRI acquisition and segmentation

**Fig. 1:**
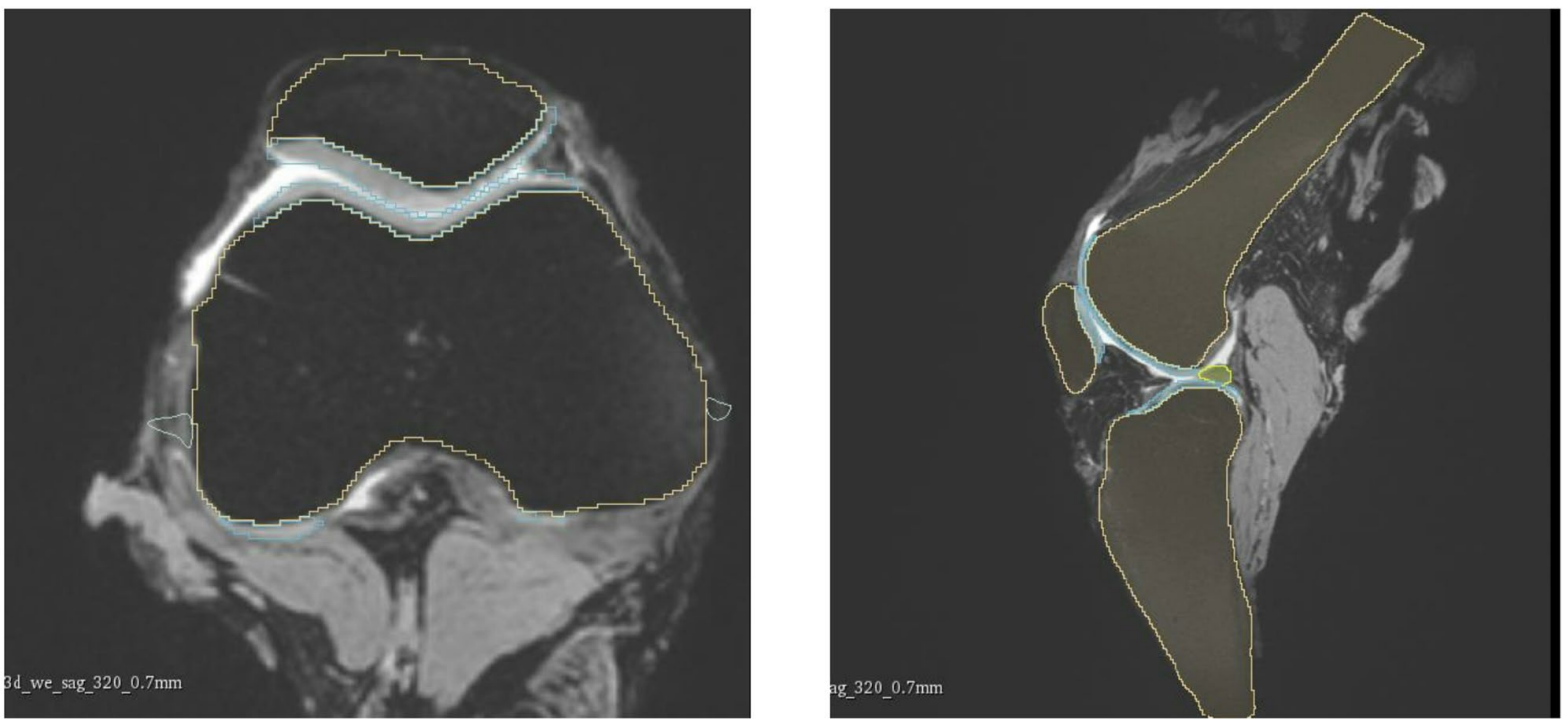
Segmentation of the MRI scans using 3D Slicer, obtaining relevant osseous and cartilaginous structures

**Fig. 2:**
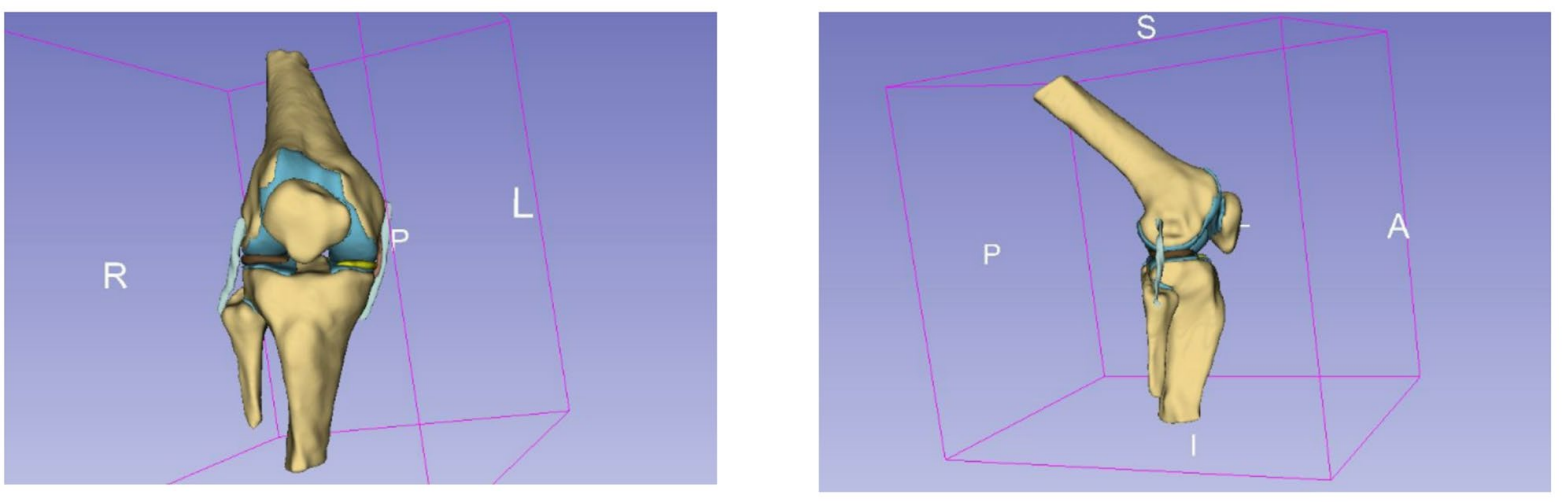
3D slicer, segmented knee model in ventral view (left) and sagittal view (right)

**Fig. 3:**
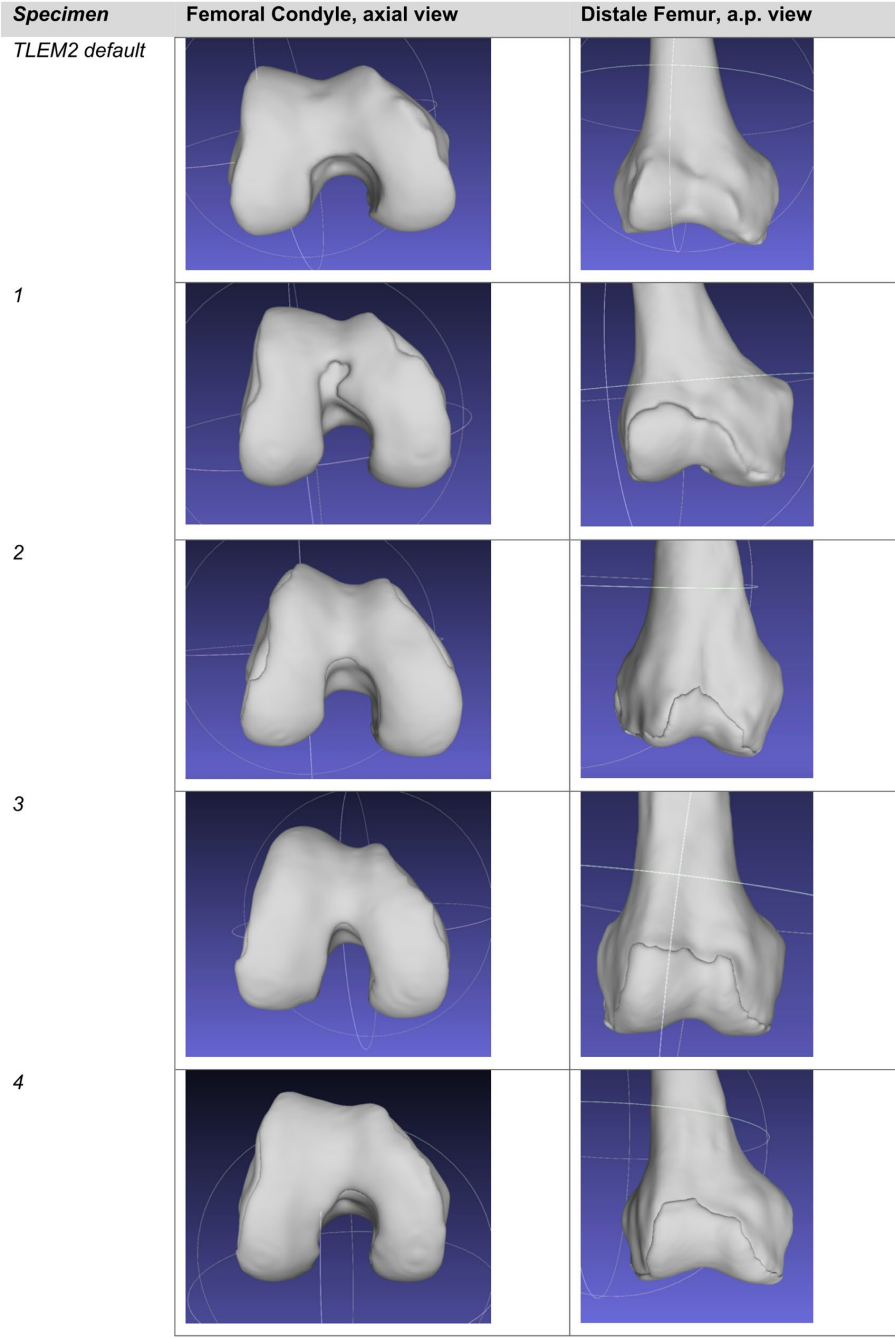
3D models of the knee specimen

Four human cadaveric lower limbs (1 female, 3 male; mean age 51.3 ± 15.0 years; body mass 72.3 ± 14.1 kg; height 175.3 ± 7.1 cm) were imaged using magnetic resonance imaging (MRI) as part of an established anatomical dataset. The limbs originated from healthy adult donors. Each scan encompassed approximately 20 cm of the distal femur, 22 cm of the proximal tibia, and the complete patella. Imaging data were exported in DICOM format and segmented using 3D Slicer (version 4.10.2; BWH and the Slicer Community, USA) to extract relevant osseous and cartilaginous structures, shown in Figs. 1 and 2. The segmented volumes were converted into three-dimensional surface meshes and exported in Standard Tessellation Language (STL) format for model integration, shown in Fig. 3.

Ethical approval was given by the Ethics committee of the University of Munich (23–0587).

### 3D model integration and implementation

Segmented femur, tibia, and patella geometries were processed in Autodesk Meshmixer and CATIA V5 to enable integration into the AnyBody Modeling System. Cartilage surfaces were embedded into the bony meshes to preserve joint functionality. Personalized segments were aligned with generic AMMR templates using anatomical landmarks, and merged with smoothed transitions [12]. Coordinate mapping was performed in CATIA V5, and reference points were implemented via custom AnyScript routines to replace default bones with subject-specific STL geometries in the simulation model.

### Musculoskeletal modeling and simulation protocol

The subject-specific models were implemented into the AMMR v2.2.2 using the TLEM2-based “SquatModel [12]” Personalization was applied unilaterally and mirrored to the contralateral limb to ensure symmetry. Simulations involved a loaded squat task to 90° of knee flexion at a controlled angular velocity of 60°/s. Output parameters included joint reaction forces and muscle force of the quadriceps.

The original TLEM2 patellofemoral joint mechanism was preserved. While the bony trochlear geometry was replaced with subject-specific surfaces (generic templates), the mechanical definition of the joint remained simplified and was not based on contact mechanics or deformable cartilage models. Patellofemoral forces were calculated as the resultant joint reaction forces according to ISB recommendations [13]. Specifically, the mediolateral component of the patellofemoral joint reaction force was defined as the force acting along the local x-axis, directed medial to lateral. This coordinate system followed ISB recommendations, as described by Derrick et al. [13], with forces decomposed accordingly using AnyBody output variables.

The tibiofemoral joint was modeled with a single rotational degree of freedom (flexion/extension), with all translational movements constrained, in accordance with the TLEM2-based “SquatModel” in AMMR v2.2.2. The patellofemoral joint mechanism followed the predefined kinematic coupling provided in the AMMR, where patellar translation and tilt are determined as a function of knee flexion angle. No additional translational or rotational degrees of freedom were assigned to the patella, and joint motion was not governed by dynamic contact interactions or ligamentous constraints. As such, patellar tracking was geometrically prescribed rather than force-resolved.

### Simulation protocol

**Fig. 4:**
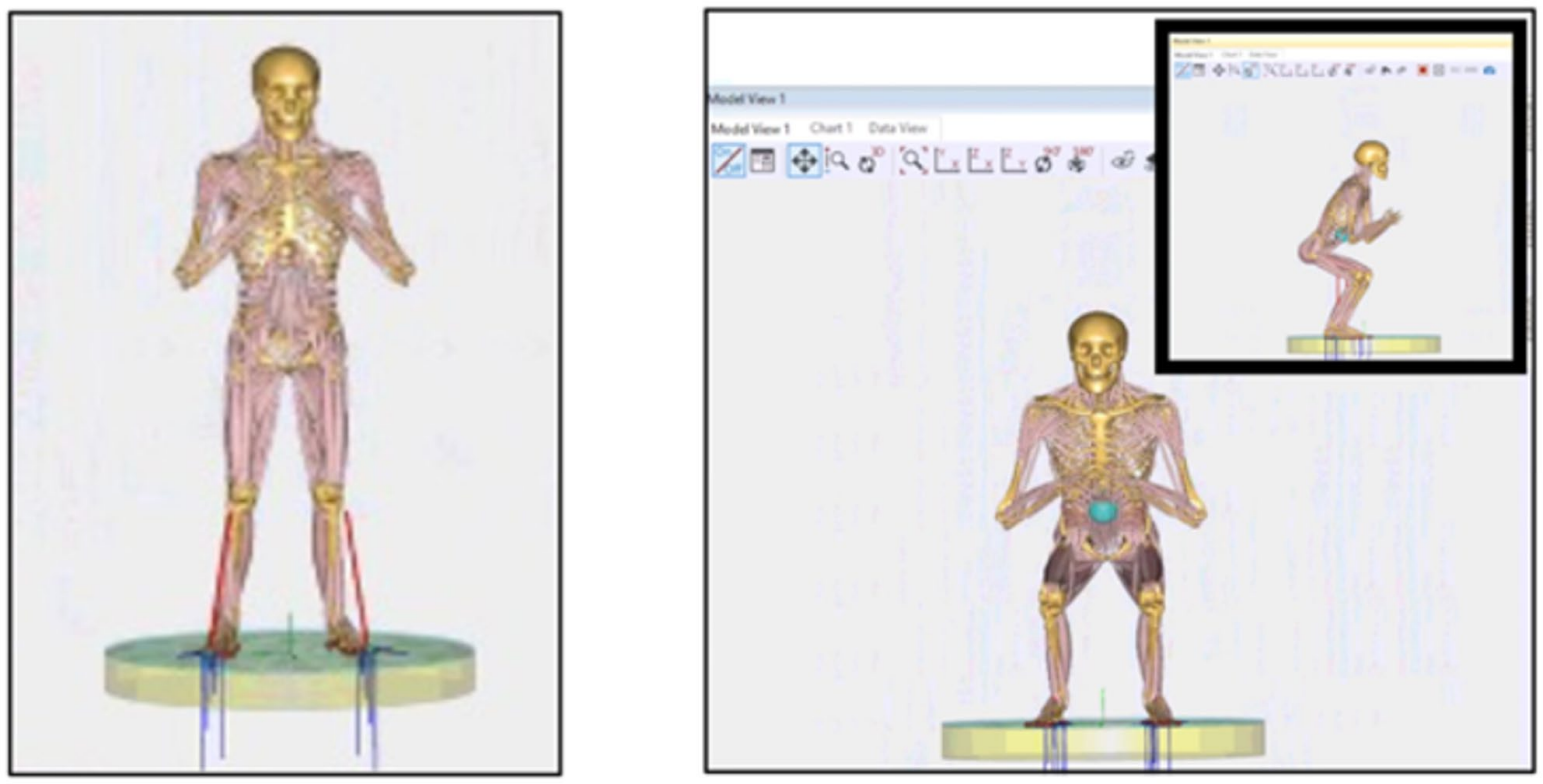
Representation of the squat in the individualized musculoskeletal model in AnyBody (*Squat.main*)

The musculoskeletal simulation was driven by a predefined squat trajectory implemented via a kinematic driver in the AnyBody Modeling System (AMMR v2.2.2), as used in previous studies using the TLEM2 dataset [14, 15]. The motion consisted of a symmetric knee flexion-extension cycle between 0° and 90° of flexion at a constant angular velocity of 60°/s. The hip and ankle joints followed the default kinematics from the TLEM2-based “SquatModel”. The simulated squat setup and subject-specific model integration are illustrated in Fig. 4.

No experimental motion data (e.g., marker trajectories or inverse kinematics) were used; the movement was fully computer-driven based on a scripted angular profile. This approach is commonly used in simulation environments where standardized joint kinematics are required to isolate anatomical influences on loading patterns [14, 16, 17]. Foot-ground contact was modeled using fixed foot constraints and ground reaction force prediction was used according to Fluit et al. and Jung et al. [14, 18].

The term “loaded” refers to internally simulated bodyweight-level forces, consistent with the standard AMMR squat configuration. No external weights were applied. Joint reaction forces and muscle forces were estimated via inverse dynamics using the standard Hill-type muscle model implemented in AnyBody [15, 16]. No EMG data were used for calibration or validation.

Patellofemoral joint reaction forces were normalized to bodyweight (N/kg BW). All simulations were performed bilaterally, with personalized knee geometry applied to one side and mirrored to the contralateral limb to preserve symmetry.

The musculoskeletal model was based on the validated AMMR framework, which has been previously used in similar lower limb simulations to investigate joint loading, muscle coordination, and implant mechanics under standardized movement conditions [14, 17].

## Results

**Fig. 5:**
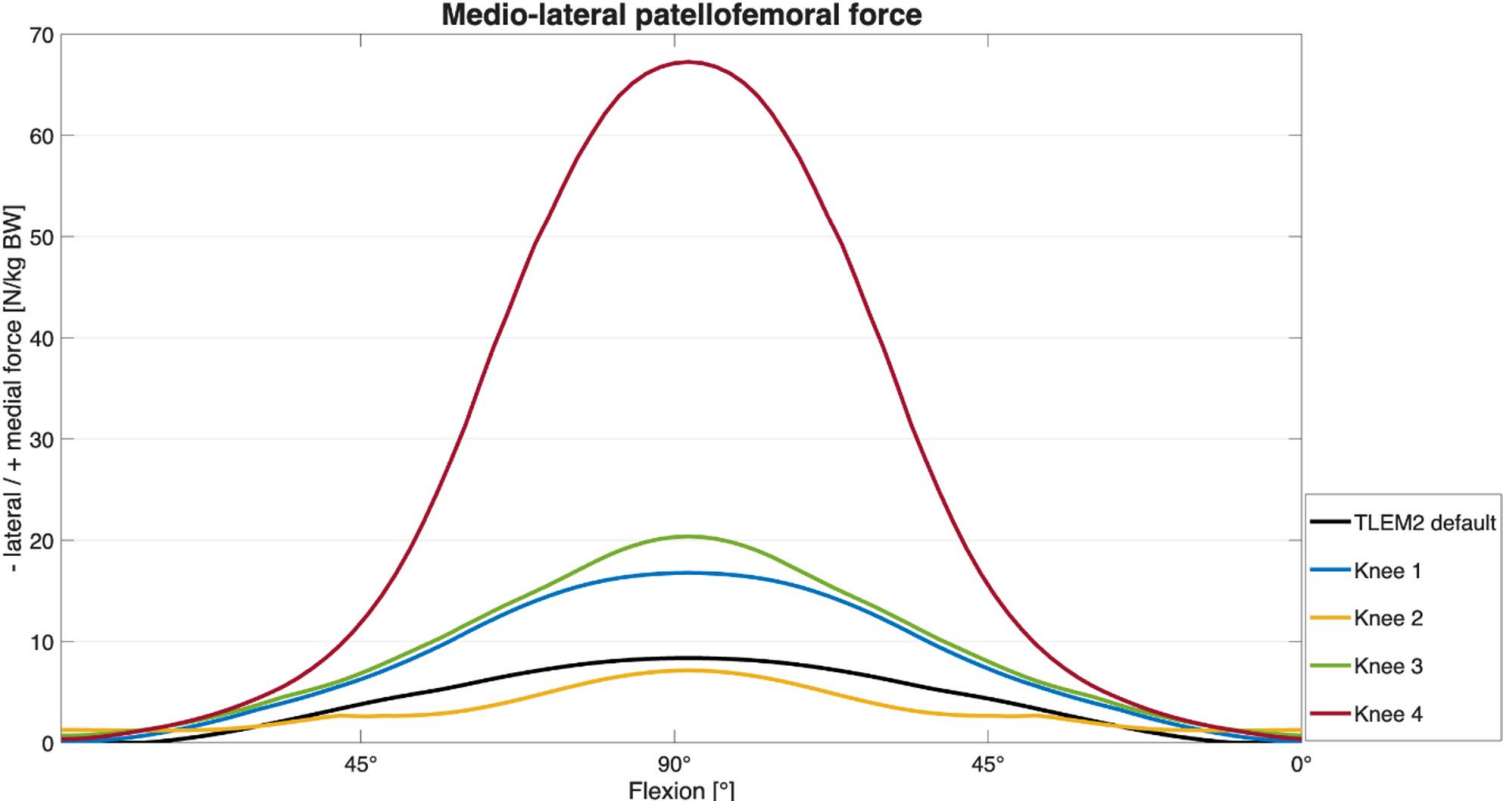
The mediolateral patellofemoral force normalized to body weight (BW) during knee flexion up to an angle of 90°, comparing the default unmodified model (“TLEM2 default”) with each of the four individualized knee models (“Knee 1–4”)

Figure 5 illustrates the mediolateral patellofemoral force during knee flexion up to an angle of 90°, comparing the default unmodified model (“TLEM2 default”) with each of the four individualized knee models (“Knee 1–4”). The unmodified TLEM2 geometry is provided in the AMMR and used here as a baseline comparison.

The data are based on simulation results from the four personalized models. The mediolateral joint reaction force increased progressively with knee flexion, reaching its peak at 90° of flexion. During subsequent knee extension, a comparable decrease in force was noted. On average, a maximum of 24 (± 25) N/kg BW with a range from 7 N/kg BW to 67 N/kg BW was achieved (see Table 1).

**Table 1:** Mediolateral patellofemoral joint reaction force during knee flexion

Specimen	Maximum mediolateral patellofemoral joint reaction force in Newton/kilogram body weight [*N*/kg BW]
TLEM2 default	8
1	17
2	7
3	20
4	67
Mean peak force	24 (± 25) *N*

**Fig. 6:**
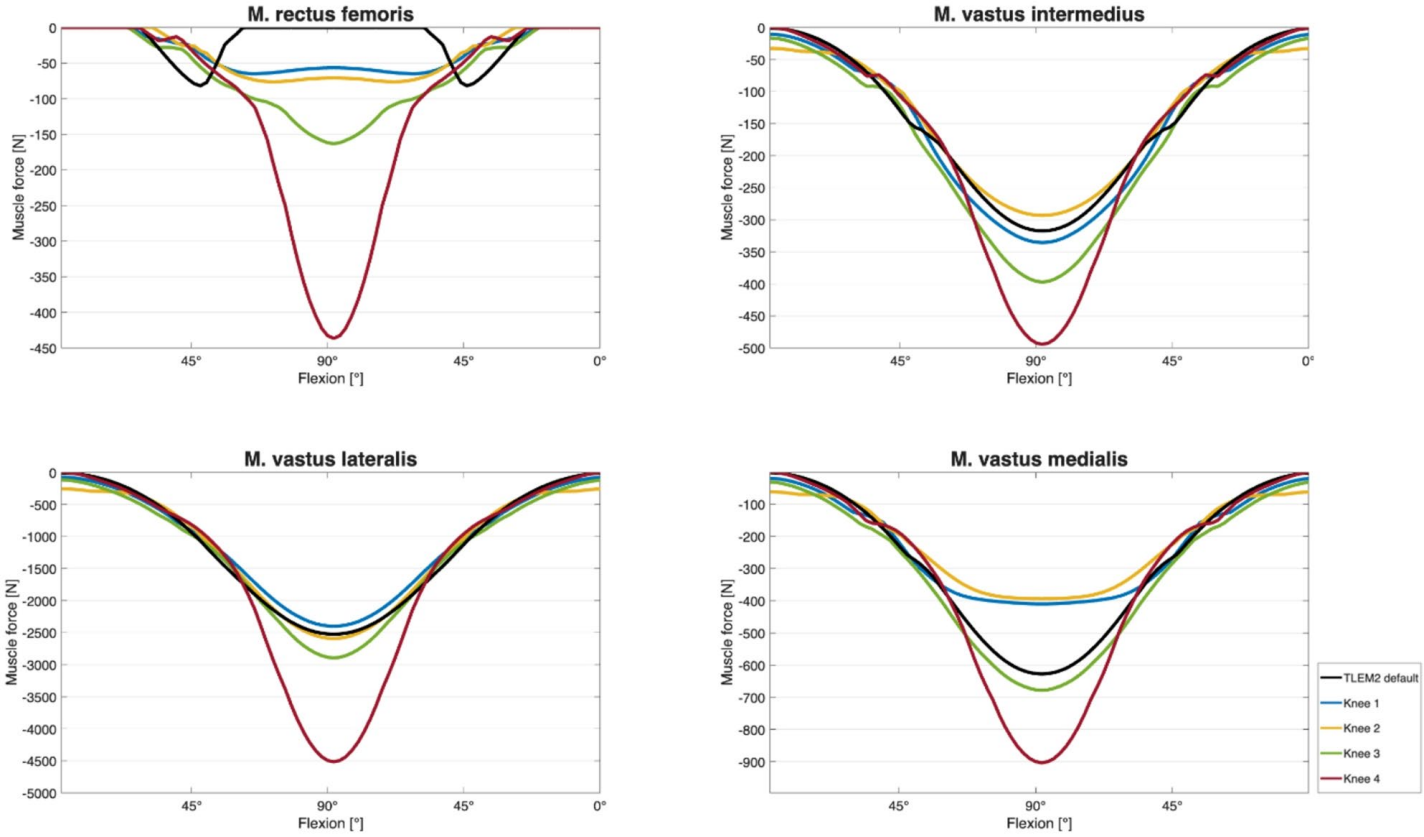
Traction force of the four parts of the quadriceps muscle during a squat- knee flexion cycle

In Fig. 6; Table 2, the muscle strength required to perform the squat movement are shown for each knee. Here too, the force required increases progressively with increasing knee flexion and shows the maximum at 90° flexion. Specimen-Knee 4, which had the highest medio-lateral patellofemoral joint reaction force, showed the highest force in the quadriceps muscle. The muscle strength curve showed symmetry between flexion and extension of the knee joint, which was expected given that the squat motion was predefined and kinematically symmetric in both directions.

**Table 2:** Estimated maximum muscle force of the M. rectus femoris, M. vastus intermedius, M. vastus lateralis, M. vastus medialis (inverse dynamics)

Specimen	M. rectus femoris in Newton [*N*]	M. vastus intermedius in Newton [*N*]	M. vastus lateralis in Newton [*N*]	M. vastus medialis in Newton [*N*]
TLEM2 default	−82	−317	−2523	−628
1	−65	−336	−2399	−410
2	−76	−293	−2591	−394
3	−163	−397	−2893	−678
4	−436	−494	−4514	−904
Mean muscle force	−164 (± 157) N	−367 (± 81) N	−2984 (± 874) N	−603 (± 211) N

In Table 2 the maximum traction force of the muscles M. rectus femoris, M. vastus intermedius, M. vastus lateralis and M. vastus medialis during a squat movement are shown.

Muscle forces are reported as magnitudes. Negative values displayed in AnyBody output represent force direction along the model’s coordinate system and do not imply negative muscle contraction. For clarity, force magnitudes are presented herein.

## Discussion

The major findings of this study are that the estimated mediolateral component of the patellofemoral joint reaction forces increase progressively with knee flexion during a loaded squat, and that these forces differ substantially depending on individual anatomical features. In our subject-specific adaptation of the squat model, the highest lateral force and quadriceps force were observed in the specimen with pronounced trochlear dysplasia (Dejour type B, Specimen 4). This observation confirms the biomechanical effect of trochlear dysplasia on PFJ loading in a standardized model, consistent with previous cadaveric and imaging studies [19, 20]. It is important to note that the reported mediolateral joint reaction forces do not represent direct patellofemoral contact pressures nor a mechanical measure of joint stability, however, they serve as valid surrogate for assessing directional loading tendencies relevant to PFJ instability. These forces represent the net effect of muscle, ligament, and joint constraints as implemented within the patellofemoral mechanism in the AMMR and should be interpreted as a surrogate indicators. These results highlight the value of MRI-based, subject-specific musculoskeletal models as a clinically relevant tool to identify patients at increased risk for patellofemoral instability and to support individualized (surgical) treatment planning.

The AnyBody Modeling System provides a library of pre-validated musculoskeletal application models, including a standardized squat model that simulates dynamic lower limb motion under loading conditions. These models are biomechanically validated and serve as robust platforms for simulating joint kinetics and kinematics.

For the purposes of this study, the generic squat model was modified to incorporate subject-specific knee joint anatomy. This personalized model was employed to simulate a loaded squat movement and to quantify the mediolateral component of the patellofemoral joint reaction force throughout the flexion cycle.

The observed mediolateral joint reaction forces ranged from 7 N/kg BW to 67 N/kg BW (mean: 24 ± 25 N/kg BW), with the greatest force observed in specimen 4. Morphological analysis of this knee revealed a supratrochlear bony prominence corresponding to a Dejour type B trochlear dysplasia (shown in Fig. 3), a condition associated with reduced patellar containment and increased lateral translation [9, 21, 22]. This is consistent with prior in vitro findings, where supratrochlear spurs led to increased lateral contact pressures and diminished patellar stability. Trochlear dysplasia has previously been shown to increase lateral patellar tracking and contact pressure, and to reduce patellar containment under load [22]. The significantly elevated quadriceps force observed in specimen 4, particularly within the M. vastus lateralis, likely represents a compensatory response to this altered trochlear morphology.

The role of bony anatomy – particularly the trochlear groove – is central to patellofemoral stability. An anatomically deep and steep trochlea offers inherent stability by guiding the patella during flexion [23, 24]. In contrast, trochlear flattening or elevation of the patella (patella alta) delays trochlear engagement, thereby increasing the risk of dislocation. In normal anatomy, the patella engages the trochlear groove at approximately 20–30° of flexion; in patella alta, this engagement occurs later, reducing the efficacy of bony stabilization [25, 26].

Another important factor is the tibial tuberosity–trochlear groove (TT–TG) distance, which, together with the Q-angle, determines the lateral vector of the patellar tendon. An increased TT–TG distance has been associated with patellar maltracking and overload in the lateral compartment [27, 28]. The integration of such anatomical features into subject-specific models, as demonstrated here, offers a detailed approach to understanding joint mechanics and pathological loading conditions.

Beyond trochlear morphology, the position of the patella and the alignment of the extensor apparatus are major contributors to patellofemoral biomechanics. In particular, patella alta delays trochlear engagement and increases the risk of lateral dislocation by reducing bony constraint in early flexion [21]. Similarly, the tibial tuberosity–trochlear groove (TT–TG) distance influences the Q-angle and, consequently, the lateral force vector acting on the patella [27, 28]. Although these parameters were not systematically varied in the current study, the model architecture allows for programmable adjustments, for example to both TT–TG distance and patellar height. This opens the possibility for future investigations simulating pathologies such as excessive lateralization or patella alta, as well as the biomechanical consequences of surgical interventions like tibial tubercle transfer or patellar distalization.

The present study also contributes methodologically by offering a non-invasive alternative to in vivo or in vitro testing. By embedding individualized osseous and cartilaginous structures into a biomechanically validated simulation framework, it becomes possible to investigate complex interactions between soft tissue forces and joint morphology. This approach is particularly valuable for evaluating hypothetical surgical corrections, such as trochleaplasty or tibial tubercle transfer, prior to clinical application.

The ability to model such structural variations in a controlled, subject-specific environment represents a valuable step toward improving individualized treatment strategies. The model may serve as a preoperative assessment tool to simulate individual anatomical configurations and their biomechanical consequences, ultimately aiding surgical decision-making. By modifying bony input geometries and force parameters, one can systematically analyze the effects of isolated or combined anatomical risk factors on patellar tracking, joint loading, and muscular compensation. This holds particular promise for preoperative planning in patients with recurrent patellar instability, where surgical decision-making often depends on subtle morphological features that are difficult to interpret in static imaging alone.

A further strength of this modeling approach lies in its potential to reduce reliance on in vivo or cadaveric experiments. The simulation environment enables dynamic analyses of force distributions, segmental kinematics, and joint reaction forces under realistic loading conditions. Moreover, the model can be extended to simulate more complex, instability-provoking movements, including stair descent, pivoting, or running.

Although the model does not provide direct measures of patellofemoral stability, the results illustrate how anatomical variation can influence mediolateral loading patterns during controlled motion. The markedly increased loading in the trochlear-dysplastic specimen supports previously reported associations between trochlear morphology and lateralization tendencies.

Several limitations must be acknowledged. First, the models relied on non-weightbearing cadaveric MRI Scans and computer-driven kinematics. Second, the patellofemoral joint was modeled using rigid geometries and simplified kinematic coupling without implementing dynamic contact modeling. No deformable cartilage or force-resolved contact interactions were simulated. This simplification limits the ability to capture localized contact pressures and may underestimate the influence of joint congruency, especially in cases of trochlea dysplasia. Third, muscle forces were estimated via inverse dynamics without EMG validations. Fourth, no validation of simulated outputs against experimental data such as EMG signals, ground reaction forces, or in vivo joint loads was performed. This lack of direct validation limits the interpretability of the absolute force magnitudes and necessitates cautious interpretation. However, the predicted ranges for mediolateral patellofemoral forces and quadriceps muscle forces are consistent with previously published musculoskeletal simulations of lower-limb tasks using the AMMR framework and Hill-type muscle models. Prior studies have demonstrated similar magnitudes of quadriceps loading and directional trends of patellofemoral joint reaction forces during squat-like movements. Furthermore, the simulation outputs showed physiologically plausible behavior, including progressive force increase with knee flexion and increased quadriceps demand in specimens with trochlear dysplasia. Nonetheless, future studies are warranted to validate model predictions against experimental motion capture, EMG, or in vivo fluoroscopic data to further increase the model’s accuracy and clinical applicability. Also, the muscle-tendon parameters such as optimal fiber length, pennation angle, or tendon slack length were not personalized across specimens but adopted from the TLEM2 dataset. This simplification may have influenced force estimations and should be addressed in future refinements.

Finally, although the squat represents a physiologically relevant task, further motion types will be required to generalize the findings.

The presented modeling approach offers a methodological framework for individualized biomechanical analysis, which may support future investigation into patient specific risk factors. Future studies will extend this methodology to incorporate a broader range of pathological conditions, particularly in patients with multifactorial instability driven by complex interactions between anatomical structures and dynamic stabilizers, to further enhance its clinical applicability.

## Conclusion

This study successfully demonstrated that integrating individualized MRI-based anatomy into a musculoskeletal simulation model enables quantification of subject-specific patellofemoral joint loading and muscle activation patterns. The findings underscore the influence of trochlear morphology and other anatomical risk factors on mediolateral force distribution and highlight the value of patient-specific models in preoperative assessment, treatment planning, and biomechanical research on patellofemoral instability.

## Data Availability

The data presented in this study are available on request from the corresponding authors.
